# Hypoxia Alters the Expression of Dipeptidyl Peptidase 4 and Induces Developmental Remodeling of Human Preadipocytes

**DOI:** 10.1155/2016/7481470

**Published:** 2016-01-03

**Authors:** Helena H. Chowdhury, Jelena Velebit, Nataša Radić, Vito Frančič, Marko Kreft, Robert Zorec

**Affiliations:** ^1^Laboratory for Neuroendocrinology-Molecular Cell Physiology, Institute of Pathophysiology, Faculty of Medicine, University of Ljubljana, Zaloska 4, SI-1000 Ljubljana, Slovenia; ^2^Celica Biomedical Center, Tehnološki Park 24, SI-1000 Ljubljana, Slovenia; ^3^Department of Biology, Biotechnical Faculty, University of Ljubljana, Večna Pot 111, SI-1000 Ljubljana, Slovenia

## Abstract

Dipeptidyl peptidase 4 (DPP4), a transmembrane protein, has been identified in human adipose tissue and is considered to be associated with obesity-related type 2 diabetes. Since adipose tissue is relatively hypoxic in obese participants, we investigated the expression of DPP4 in human preadipocytes (hPA) and adipocytes in hypoxia, during differentiation and upon insulin stimulation. The results show that DPP4 is abundantly expressed in hPA but very sparsely in adipocytes. During differentiation *in vitro*, the expression of DPP4 in hPA is reduced on the addition of differentiation medium, indicating that this protein can be hPA marker. Long term hypoxia altered the expression of DPP4 in hPA. In *in vitro* hypoxic conditions the protease activity of shed DPP4 is reduced; however, in the presence of insulin, the increase in DPP4 expression is potentiated by hypoxia.

## 1. Introduction

Dipeptidyl peptidase 4 (DPP4) is a 110 kDa transmembrane glycoprotein. A soluble form of DPP4 (sDPP4) in the circulation is the result of proteolytic cleavage of the membrane bound form [1]. DPP4 has a rare protease activity, cleaving N-terminal x-Pro dipeptide from selected proteins. Besides enzymatic inactivation of incretins, DPP4 also mediates degradation of several growth factors, neuropeptides, chemokines, and vasoactive peptides, which results in alterations in their biological activity, often by altering their receptor specificity [2].

Altered DPP4 activity has been reported in a number of diseases, including type 2 diabetes [3, 4] and tumor biology [2, 5–7]. It is thought to be associated with sensitivity to anticancer agents in haematologic malignancies and is involved in the development of various chronic liver diseases [8]. DPP4 was considered as a therapeutic target for type 2 diabetes as it degrades incretins: glucagon-like peptide- (GLP-) 1 and gastric inhibitory peptide (GIP). Both hormones cause an increase in insulin secretion. DPP4 inhibitors target the enzyme activity of DPP4, thus prolonging the insulinotropic effects of incretins [9].

In patients with type 2 diabetes acute hypoxia increases the glucose uptake into the tissue [10, 11]; however, prolonged exposure to hypoxia has been associated with induction of insulin resistance in adipose tissue [12, 13]. More specifically, adipose tissue hypoxia, which develops with the onset of obesity [14, 15], has been linked to the development of insulin resistance and type 2 diabetes by decreasing insulin signaling pathways [13]. It was reported that DPP4 is expressed in preadipocytes and in adipocytes [16], indicating that adipose tissue might be a major source of circulating DPP4. Therefore, an altered expression of adipose tissue DPP4 could be linked to the development of type 2 diabetes; however the factors that alter the expression of DPP4 are poorly understood.

In the present study we developed a confocal microscopy assay to study the expression of DPP4 in human preadipocytes (hPA). We evaluated DPP4 expression during the differentiation of human preadipocytes into adipocytes and studied how insulin affects the expression and activity of released DPP4. The results show that in hypoxic conditions the protease activity of shed DPP4 is reduced. Interestingly, insulin increases DPP4 expression and this is potentiated by hypoxia.

## 2. Experimental Procedures

### 2.1. Chemicals

Dulbecco's modified Eagle's medium/Ham's F12 (DMEM/F12), l-glutamine, dexamethasone, isobutylmethylxanthine (IBMX), biotin, d-pantothenic acid hemicalcium salt, phosphate-buffered saline (PBS), bovine serum albumin (BSA), trypsin-EDTA, Trypan Blue, and goat serum were purchased from Sigma (St. Louis, MO). Insulin was purchased from Novo Nordisk (Bagsvaerd, Denmark). Fetal bovine serum (FBS) was obtained from Biochrom (Berlin, Germany). Rosiglitazone maleate was obtained from GlaxoSmithKline (Worthing, UK). Dimethyl sulfoxide (DMSO) was purchased from Merck Schuchardt (Hohenbrunn, Germany). Antibiotic-antimycotic mixture was purchased from Gibco (Invitrogen Corporation, NY). Paraformaldehyde was purchased from Thermo Scientific, USA.

### 2.2. Primary Preadipocyte Maintenance and Differentiation Procedure

Preadipocytes (human subcutaneous) were purchased from ZenBio, Inc. (Research Triangle Park, NC). Cells were cultured under standard conditions (at 37°C, humidified atmosphere, 5% CO_2_) in PM medium. For each set of experiments, cells were seeded on coverslips in uniform density, which was provided by counting cells before seeding. For differentiation into adipocytes we used the protocol from ZenBio. The start of the differentiating procedure was marked as day 0. On indicated days the 16 h conditioned 1% BSA/PBS medium was collected, filtered (0.2 *μ*m), and analyzed for enzymatic activity and quantification of sDPP4. On day 21 60–70% of cells were fully differentiated, indicated by the accumulation of lipid droplets (not shown). The cells were than subjected to immunolabeling protocol or trypsinized and counted using a hemocytometer (improved Neubauer type). Three separate samples were prepared for each time point.

### 2.3. Hypoxia Treatment* In Vitro*

For the study of DPP4 expression under hypoxic conditions, preadipocytes were cultured in a hypoxic chamber (Billups-Rothenberg, Dell Mar, CA), flushed twice at a 2 h interval for 4 min with a gas mixture consisting of 1% O_2_, 5% CO_2_, and 94% N_2_, and incubated for the indicated time at 37°C in a humidified atmosphere.

### 2.4. Immunocytochemistry

To identify the expression of HIF-1*α* and DPP4 in the preadipocytes, primary monoclonal mouse anti-HIF-1*α* antibodies and primary monoclonal mouse anti-DPP4 antibodies (both Abcam, Cambridge, UK; ab8366 and ab3154) were used and secondary goat anti-mouse antibody conjugated to Alexa Fluor 546 and to Alexa Fluor 488, respectively (A11003 and A11001, Molecular Probes). Cells were washed with PBS and fixed in 2% paraformaldehyde for 20 min, which was sufficient to permeabilise the cell membrane and allow binding of antibody to a total cell protein. Cells were incubated at 37°C in blocking buffer (3% BSA, 10% goat serum in PBS) for 1 h, with primary antibodies for 2 h and with secondary antibodies for 45 min. Subsequently, they were mounted using a Light Antifade kit (Invitrogen).

### 2.5. Confocal Microscopy

Z-stacks of immunolabeled cells were acquired using a Zeiss LSM 510 confocal microscope through a Plan Apochromatic oil-immersion objective (63x, NA = 1.4), excited by the 488 nm argon laser line and filtered with the 505–560 nm low-pass emission filter and excited by the 543 nm He/Ne laser line and filtered with the 560 nm low-pass emission filter. Images were analyzed quantitatively using LSM 510 software (Carl Zeiss). Eight to 15 markers were manually set to the cell perimeter, and the software interpolated the curve between them. The area above the threshold (20% of the maximal fluorescence intensity) fluorescence intensity relative to the cell cross-sectional area was determined.

### 2.6. Enzymatic Activity

Peptidase activity of the sDPP4 released from nonpermeabilised cells from the cell surface was determined using the DPPIV/CD26 assay kit for biological samples (Enzo Life Sciences, Plymouth Meeting, PA) according to the manufacturer instructions. The relative fluorescence units for each sample were calculated by plotting the linear region of the change in fluorescence over time and calculating the slope of the line. This was then used with the conversion factor to calculate the activity expressed as pmol/min and divided by the number of cells in individual samples to obtain the values expressed as pmol/min/cell. Data are presented as means ± s.e.m. of all tests (*n* = 9).

### 2.7. ELISA

The amount of sDPP4 released by the cell at different stages of differentiation and at different oxygenation of the cell atmosphere was quantified by a Human DPPIV/CD26 Quantikine ELISA kit (R&D Systems, Minneapolis MN) following the manufacturer's recommendations.

## 3. Results

### 3.1. Hypoxia-Mediated Reduction in DPP4 Expression in Single hPA

**Figure 1:**
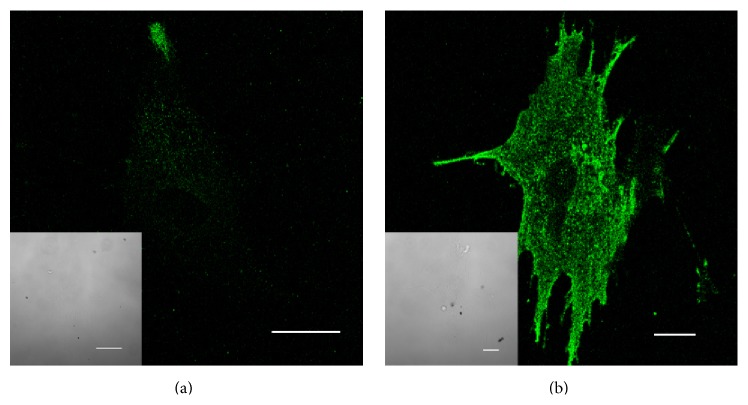
Expression of HIF-1*α*, a hypoxia marker, in human preadipocytes cultured* in vitro*. Confocal images and transmission light images (insets) of human preadipocytes immunolabeled with antibody against HIF-1*α*. Preadipocytes were cultured for 2 days in normoxic conditions (a, 18% O_2_) or hypoxic conditions (b, 1% O_2_). Cells were fixed and immunolabeled with an antibody against HIF-1*α*. Inserts show transmission light microscopy images of the same cells as in the confocal fluorescent images. Scale bars indicate 20 *μ*m.

hPA were cultured in a normoxic chamber (18% pO_2_) and in a hypoxic chamber (1% pO_2_). To confirm that these hPA responded to hypoxia, cells were immunolabeled with antibodies against hypoxia-inducible factor-1*α* (HIF1-*α*). The results show that exposure to 1% pO_2_ induced a massive expression of transcriptional factor HIF1-*α* (Figure 1(b)) and are consistent with previously published data [17]. In cells cultured in 18% pO_2_, the expression of HIF1-*α* was negligible (Figure 1(a)). Thus hPA incubated under hypoxic conditions responded physiologically to the lowered pO_2_.

**Figure 2:**
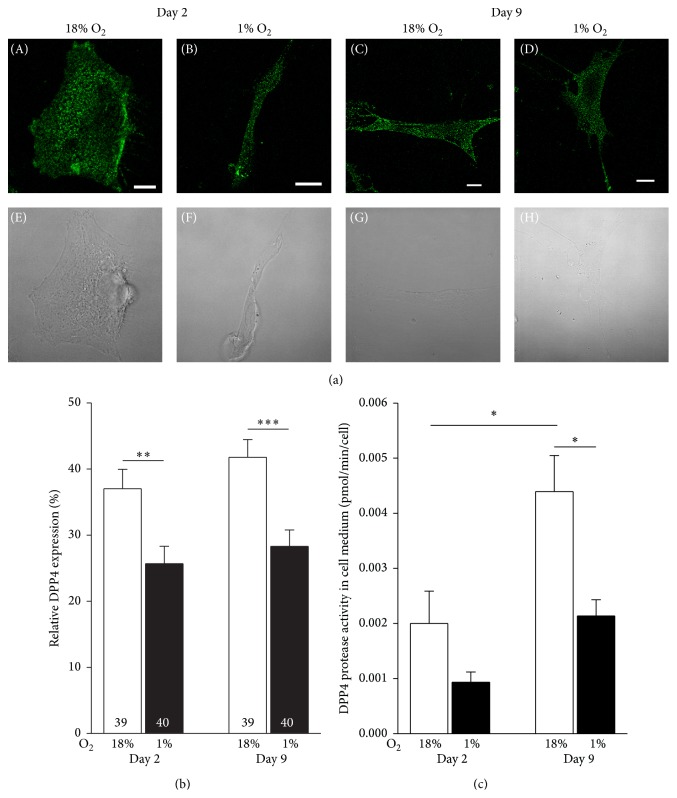
Oxygenation-dependent expression density and shedding of DPP4 in human preadipocytes cultured* in vitro*. (a) Confocal images (upper panels) and transmission light microscopy images (lower panels) of human preadipocytes (subconfluent cultures) immunolabeled with antibody against DPP4. Each panel shows representative confocal images of a cell incubated for 2 days (A, B, E, and F) and 9 days (C, D, G, and H) under normoxic (A, C, E, and G) and hypoxic (B, D, F, and H) environmental conditions. Scale bars indicate 10 *μ*m. (b) Relative expression density of DPP4 in human preadipocytes cultured for 2 and 9 days in normoxic or hypoxic conditions. Numbers denote the number of cells imaged. (c) Normalized DPP4 protease activity in conditioned medium. Asterisks denote statistically significant difference (^*∗*^ *P* < 0.05; ^*∗∗*^ *P* < 0.01; ^*∗∗∗*^ *P* < 0.001, two-way ANOVA).

To examine the expression of DPP4 in a hypoxic environment* in vitro* and to get insights into short and long term effects of hypoxia, cells were incubated at 1% pO_2_ for 2 and 9 days and labeled with the anti-DPP4 antibody. The images of the largest optical slice of hPA (Figure 2(a)) were analyzed by determining the percent area of DPP4-labelled part relative to the area of the entire optical slice of a cell. Note that the staining pattern of cells in Figure 2(a) is evenly distributed through the entire cell area. The 2-day exposure to hypoxia significantly reduced the expression density of DPP4 to 25.7 ± 2.7% compared with cells in normoxia (37.0 ± 2.9%; *P* < 0.001; Figure 2(b)). Prolonged incubation (9 days) had no further effect on DPP4 expression density in a hypoxic (28.3 ± 2.5%) or normoxic environment (41.8 ± 2.7%).

### 3.2. Time-Dependent Increase in the Protease Activity of sDPP4 Is Reduced by Hypoxia

DPP4 is a transmembrane protein; however, it is also active in its soluble form, after shedding the extracellular domain of the protein from the cell's surface. We investigated the protease activity of sDPP4 in conditioned medium of cells incubated in different oxygenation environments. Significant differences were detected between samples incubated in normoxia for 2 versus 9 days (Figure 2(c)). After 2 days, the activity of DPP4 was 2.0 ± 0.6 fmol/min/cell, increasing to 4.4 ± 0.7 fmol/min/cell after 9 days (*P* < 0.05). In samples incubated in hypoxia, the activity also appeared to increase from day 2 to day 9, from 0.9 ± 0.2 fmol/min/cell to 2.1 ± 0.3 fmol/min/cell (*P* = 0.08). In hypoxia on day 2, the DPP4 activity was similar to that in normoxia. However, in the 9-day samples, the activity of DPP4 significantly decreased with hypoxia (*P* < 0.05). We conclude that although the amount of DPP4 protein that is shed from the cell surface of hPA is insensitive to 2 or 9 days of culture under normoxic and hypoxic conditions, we detected a significant time-dependent increase in DPP4 protease activity in normoxic controls; the increase was relatively reduced by hypoxia.

### 3.3. Differentiation-Mediated Decrease in DPP4 Expression in Adipocytes Is Potentiated by Hypoxia

**Figure 3:**
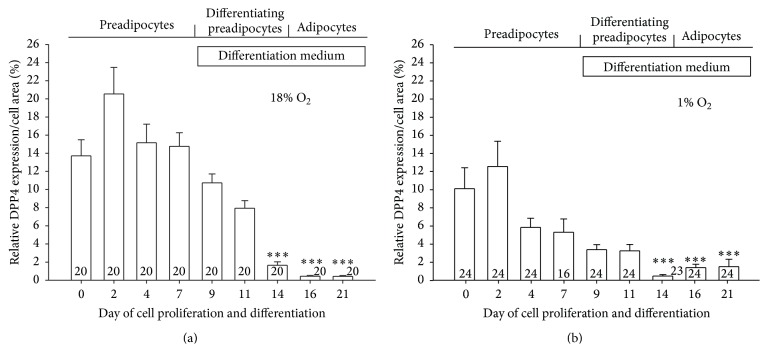
Differentiation-dependent expression density of membrane DPP4 of human preadipocytes and adipocytes cultured* in vitro* under different environmental conditions. Relative expression density of DPP4 in human preadipocytes in the course of differentiation to mature adipocytes cultured in normoxia (a) and hypoxia (b). Data are presented as mean values ± SE. Numbers denote the number of cells imaged (^*∗∗∗*^ *P* ≤ 0.001 versus preadipocytes on day 0; one-way ANOVA on ranks). On day 0, cells were already confluent.

We studied the influence of the state of differentiation of adipocytes on the DPP4 expression pattern. In normoxia DPP4 expression is variable but tends to decline as a function of time (Figure 3(a)), especially at the induction of differentiation, and is barely detectable on mature adipocytes on day 21 (Figure 3(a)). In hypoxia (Figure 3(b)), the expression of DPP4 also decreased continuously. Comparison of differentiation-dependent DPP4 expression between both oxygenation conditions revealed that, with the exception of day 21, DPP4 expression density is significantly lower under hypoxic conditions at all stages of differentiation (*P* < 0.05), consistent with data in Figure 2.

**Figure 4:**
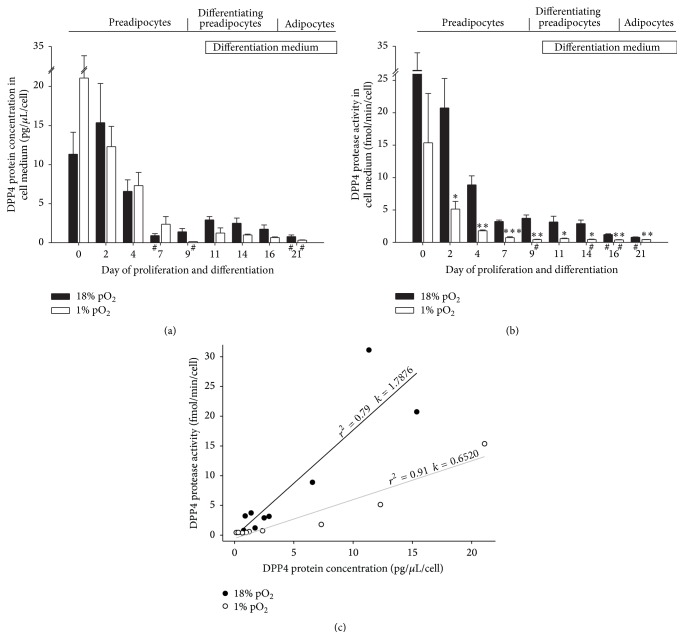
Differentiation and hypoxia reduce the shedding of DPP4 from human preadipocytes cultured* in vitro*. (a) Normalized DPP4 protein concentration in conditioned medium of cells at different stages of differentiation from preadipocytes to mature adipocytes cultured under normoxic (black bars) and hypoxic (white bars) environmental conditions. (b) Normalized DPP4 protease activity in conditioned medium of cells at different stages of differentiation from human preadipocytes to mature adipocytes cultured in normoxic (black bars) and hypoxic (white bars) environmental conditions. Data are normalized to the total cell number in the sample and presented as mean values ± SE. Asterisks above the white bars denote significant difference between hypoxic versus normoxic condition on the same differentiation day (Student's *t*-test; ^*∗*^ *P* < 0.05; ^*∗∗*^ *P* < 0.01; ^*∗∗∗*^ *P* < 0.001); # under the bars denotes significant difference compared with day 0 for respective oxygenation condition (one-way ANOVA; ^#^ *P* ≤ 0.01). (c) Correlation between the concentration of DPP4 in the conditioned media and its activity under normoxic (black dots) and hypoxic (open dots) conditions (*k*, slope coefficient; *r* ^2^, correlation coefficient). The equation of regression lines is as follows: DPP4 activity [fmol/min/cell] = (1.79 ± 0.35) × DPP4 concentration [pg/*μ*L/cell] − (0.2 ± 2.4) [fmol/min/cell] for the correlation in normoxia and DPP4 activity [fmol/min/cell] = (0.65 ± 0.08) × DPP4 concentration [pg/*μ*L/cell] − (0.6 ± 0.7) [fmol/min/cell] for the correlation in hypoxia. The slopes are significantly different (*P* < 0.001), whereas the intercepts are similar and not different from 0.

We also studied the concentration of sDPP4 in conditioned medium of cells, which tended to decrease as a function of differentiation (Figure 4(a)). With only a few exceptions (see # on Figure 4(a)), the concentration of sDPP4 seemed to decrease at a similar rate under normoxic and hypoxic conditions during differentiation.

Investigation of the protease activity of sDPP4 in the culture medium revealed that as in Figure 4(a) a similar trend of decrease during differentiation was found (Figure 4(b)). To confirm that the protease activity of sDPP4 is significantly lower under hypoxic versus normoxic conditions, we correlated the concentration of sDPP4 protein and its protease activity in normoxic and hypoxic conditions (Figure 4(c)). The slopes of the regression lines are significantly different, indicating that hypoxic conditions significantly enhance the differentiation-dependent reduction of sDPP4 protease activity, relative to the protein content of DPP4.

### 3.4. Insulin Enhances the Shedding and Protease Activity of sDPP4 in Normoxic and Hypoxic Preadipocytes

**Figure 5:**
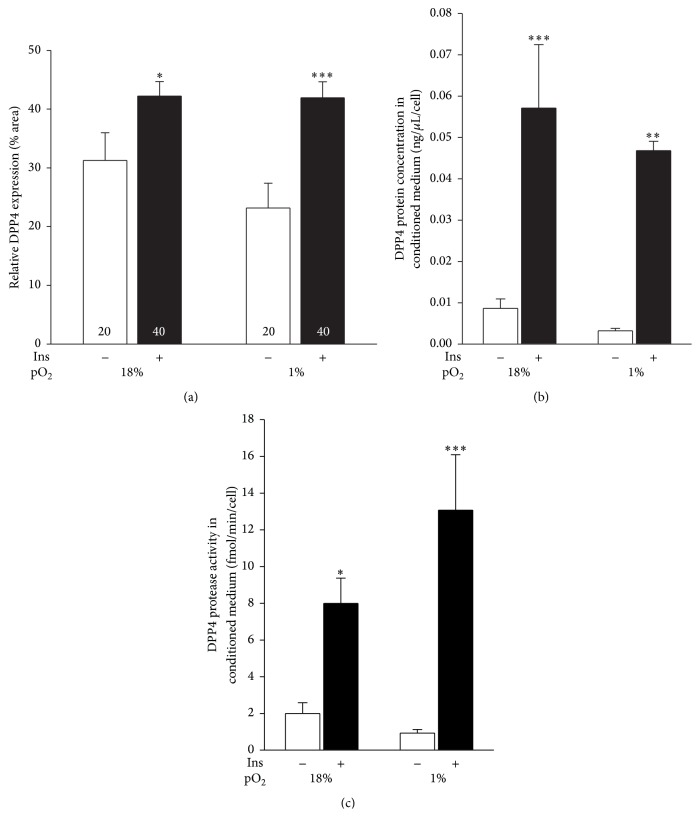
The effect of insulin on DPP4 expression and activity in human preadipocytes. (a) Human preadipocytes cultured for 2 days under normoxic (18% pO_2_) and hypoxic (1% pO_2_) conditions were treated for 30 min with 100 nM insulin (black bars). Control cells were treated with vehicle only for 30 min (white bars). Subsequently, the cells were immunolabeled with an antibody against DPP4 and imaged on a confocal microscope. The expression density of immunolabeled DPP4 was analyzed. Data are presented as means ± SE. Numbers denote the number of cells analyzed. The conditioned media were collected from cells treated for 30 min with insulin (Ins) and the protein concentration (b) and protease activity (c) of DPP4 were analyzed. Data are normalized to the number of cells in the sample and presented as means ± SE. Asterisks denote statistically significant differences compared with controls treated with vehicle only, as denoted (^*∗*^ *P* < 0.05; ^*∗∗*^ *P* < 0.01; ^*∗∗∗*^ *P* < 0.001; ANOVA).

Preadipocytes incubated for 2 days at 18% and 1% pO_2_ were stimulated with insulin (100 nM) for 30 min. In normoxia, insulin significantly increased DPP4 expression in preadipocytes (42.2 ± 2.5%; Figure 5(a)) versus stimulation with vehicle only (31.3 ± 4.7%; *P* < 0.05). A similar increase was observed in hypoxia; insulin significantly increased DPP4 expression (41.9 ± 2.7%) versus controls (23.2 ± 4.2%; *P* < 0.001).

To study shedding, we also examined the concentration of sDPP4 in the conditioned medium (Figure 5(b)) of cells stimulated with insulin. Hypoxia did not influence the sDPP4 concentration in controls or insulin-stimulated cells. However, insulin treatment significantly increased DPP4 protein shedding in normoxia (*P* < 0.001) and in hypoxia (*P* < 0.01). Under both oxygenation conditions, we recorded a pronounced effect of insulin treatment on sDPP4 protease activity in the culture medium (Figure 5(c)). In normoxia, insulin significantly increased protease activity (8.0 ± 1.4 fmol/min/cell) compared to nonstimulated cells (2.0 ± 0.6 fmol/min/cell; *P* < 0.05). In hypoxia, insulin stimulation also increased the activity of shed protein (13.1 ± 3.0 fmol/min/cell) compared to controls (0.9 ± 0.2 fmol/min/cell; *P* < 0.001). These results indicate that hypoxic conditions increase insulin-mediated expression, shedding, and protease activity of DPP4 in preadipocytes.

## 4. Discussion

### 4.1. Is DPP4 a Marker for Differentiation Status of Adipocytes?

DPP4 is abundantly expressed in hPA, while in mature adipocytes we found very modest, if any, expression of DPP4 (Figure 3). The significantly lower expression of DPP4 in mature adipocytes could not be explained with differences in cell size between both stages of differentiation, since the difference is not statistically significant (not shown). These results suggest that during differentiation from hPA into adipocytes cells gradually suppress the DPP4 expression. If this reduction in DPP4 in hPA is associated with differentiation into adipocytes* in vivo*, then DPP4 may well represent a marker for differentiation status. Therefore, like Pref-1, a stemness marker for preadipocytes, DPP4 is also robustly expressed in hPA: the higher the expression of DPP4 in preadipocytes, the higher the stem-like character of these cells. In support of this, DPP4 is abundantly expressed in cultured hPA (Figure 2), and during differentiation DPP4 expression declines (Figure 3). It is unlikely that DPP4 downexpression during differentiation is associated with a response to some of the factors in the differentiation medium added, as the DPP4 downexpression started prior to the addition of differentiation medium (Figures 3 and 4), at a stage when cells reached confluence. Although these results contrast with the report where cancer cells were studied by Abe et al. [18], the most probable explanation for the reduction of DPP4 in the (pre)adipocytes is the cell-to-cell contact-induced differentiation process into mature adipocytes that contain very modest amounts of DPP4.

DPP4 was found to be expressed in a variety of cell types [19, 20], from which it is also shed and sDPP4 may influence the shedding of membrane bound DPP4 from preadipocytes that were investigated in this study.

Current experiments revealed that the stage of differentiation of hPA into adipocytes can be assessed by monitoring the DPP4 density in cells, which can be considered as a developmental or differentiation marker, reporting the relatively undifferentiated stage of these cells. Consistent with this, it has recently been demonstrated that hypoxia inhibits adipogenesis through the HIF1*α* pathway [21, 22]. Hypoxia arrests preadipocytes in an undifferentiated state, thereby maintaining their stemness [23, 24].

The reduction in DPP4 in single differentiating cells is further supported by the determination of the concentration of sDPP4 and its enzymatic activity. Both parameters exhibit a differentiation-dependent decrease (Figures 4(a) and 4(b)) and this is consistent with the view that the soluble protein arises from the shedding of DPP4 from the plasma membrane. DPP4 has been shown to move from the cytoplasm to the cell surface rapidly and consequently the protein amount on the cell membrane is steadily proportional to the total cell protein [25]. The correlation between the concentration of sDPP4 and its activity (Figure 4(c)) shows that in hypoxia the activity is reduced by almost a factor of three relative to the concentration of soluble protein. One possible explanation would be that hypoxia lowers pH of cells. DPP4 enzymatic activity is pH dependent with the optimum at 7.8 [26]. A lower pH in hypoxic conditions would result in lower DPP4 activity. However, the activity assay was performed with buffered solution maintaining constant pH during the assay; therefore we can exclude the pH influence at the time of measurements of enzymatic activity. Although pH was not quantitatively measured during hypoxia it was observed qualitatively with pH indicator in the cell medium with no obvious changes during incubation in hypoxia, indicating that pH of the cell medium did not change significantly.

Hypoxia-related increase of preadipocyte stemness could be an additional mechanism in the development of insulin resistance. The results imply that in obesity-related hypoxia DPP4 is abundantly expressed, contributing to the reduction in insulin activity and thereby to the onset of insulin resistance in these subjects.

### 4.2. Insulin Increases DPP4 Expression Density in hPA

Most gastrointestinal (GI) hormones involved in satiety regulation and glucose metabolism, especially through regulation of insulin secretion, are hydrolyzed and inactivated by DPP4 [27]. Thus DPP4 action reduces the amount of insulin secretion. This mechanism of action is well exploited by the development of a class of antidiabetic drugs that inhibit DPP4. But little is known about how insulin influences the expression of DPP4. The results of this study demonstrate that at single-cell level insulin significantly increases DPP4 expression in hPA in a rapid manner (30 min) by about 35%. Under hypoxic conditions, this increase was 63% relative to control (Figure 5(a)). These results indicate that DPP4 is also regulated by insulin but unlike hypoxia, which renders DPP4 expression, the rapid effect of insulin is most likely posttranscriptional.

Insulin induces DPP4 expression on cells which in turn deactivated GI hormones, thereby inhibiting the stimulation of insulin secretion. In agreement with this, incubation of cells with insulin for 5 and 24 h resulted in decreased DPP4 expression to baseline level (not shown). This is probably due to insulin receptor internalization and deactivation of insulin signaling. As expected, sDPP4 in culture medium and its protease activity also significantly increased after 30 min of insulin action in both oxygenation environments.

## 5. Conclusions

This study shows that human preadipocytes express DPP4 abundantly and this expression decreases in the course of differentiation into mature adipocytes. Therefore, DPP4 can be considered a differentiation marker highlighting the stemness properties of preadipocytes. The strong inhibition of DPP4 protease activity by hypoxia and the insulin-mediated increase in DPP4 indicate that DPP4 represents an important marker for early detection of insulin resistance.
